# Synthesis of a Grease Thickener from Cashew Nut Shell Liquor

**DOI:** 10.3390/molecules28227624

**Published:** 2023-11-16

**Authors:** Son A. Hoang, Khanh D. Pham, Nhung H. Nguyen, Ha T. Tran, Ngoc Hoang, Chi M. Phan

**Affiliations:** 1 Institute of Materials Science, Vietnam Academy of Science and Technology, Hanoi 11355, Vietnam; khanhpd@ims.vast.ac.vn (K.D.P.); nhungnh@ims.vast.ac.vn (N.H.N.); 2 Vietnam Academy of Science and Technology, Graduate University of Science and Technology, Hanoi 11355, Vietnam; 3 Viet Tri Industry Institute, Viet Tri 290000, Vietnam; tranthithuhacttnpt@gmail.com; 4 Government Office, Hanoi 11355, Vietnam; hoangngocbachkhoa@gmail.com; 5 Discipline of Chemical Engineering, Curtin University, GPO Box U1987, Perth, WA 6845, Australia

**Keywords:** cashew nut shell liquor, anacardic acid, thickener, lubricant, grease, lithium hydroxide

## Abstract

Thickener, also known as a gelling agent, is a critical component of lubricating greases. The most critical property of thickener, temperature resistance, is determined by the molecular structure of the compounds. Currently, all high-temperature-resistant thickeners are based on 12-hydroxystearic acid, which is exclusively produced from castor oil. Since castor oil is also an important reagent for other processes, finding a sustainable alternative to 12-hydroxystearic acid has significant economic implications. This study synthesises an alternative thickener from abundant agricultural waste, cashew nut shell liquor (CNSL). The synthesis and separation procedure contains three steps: (i) forming and separating calcium anacardate by precipitation, (ii) forming and separating anacardic acid (iii) forming lithium anacardate. The obtained lithium anacardate can be used as a thickener for lubricating grease. It was found that the recovery of anacardic acid was around 80%. The optimal reaction temperature and time conditions for lithium anacardate were 100 °C and 1 h, respectively. The method provides an economical alternative to castor and other vegetable oils. The procedure presents a simple pathway to produce the precursor for the lubricating grease from agricultural waste. The first reaction step can be combined with the existing distillation of cashew nut shell processing. An effective application can promote CNSL to a sustainable feedstock for green chemistry. The process can also be combined with recycled lithium from the spent batteries to improve the sustainability of the battery industry.

## 1. Introduction 

Utilizing agricultural waste in chemical processes is a practical strategy to reduce CO_2_ emissions [1], with numerous economic and environmental benefits for many countries. This study focuses on the potential conversion of agricultural waste from cashew production into a special chemical for lubricating grease, which is employed widely in machinery and vehicles. 

Grease is a special group of lubricants that play an essential role in the machinery and automobile industry. Grease can be defined as semi-solid or thixotropic gels [2]. In machinery applications requiring a lubricant to maintain its original position, the lubricating grease is more effective than other liquid lubricants [3]. Chemically, lubricating grease consists of three components: base oil, thickener (also known as a gelling agent) and additives. The thickener is the most critical component, with a limited supply among the three components. The primary role of the thickener is to increase the viscosity of the base oil to a semi-solid product (grease). The thickener helps to retain the base oil in applications where a liquid lubricant would not stay in place. Since most grease applications are within the moving parts of the automotive industry, which are heated up quickly, stability at higher temperatures is a critical criterion for thickener selection. 

Chemically, the thickeners are the salts of organic acids. The salts can be simple soap (created from a single fatty acid) or complex soap (created from a mixture of acids). Historically, metals such as sodium, aluminium, calcium and lithium have been used for grease thickeners. Amongst these cations, lithium provides the highest qualities. In particular, lithium grease has the highest dropping temperature (177–204 °C) [3]. The properties are linked to the atomic nature of lithium ions, which are the smallest and most reactive. As a result, lithium-based soap accounts for about 80% of commercial high-temperature thickeners [2]. In addition to the metal, the temperature resistance of these thickeners is determined by the molecular structure of the acid. 

Currently, the two main acids for grease thickeners are long-chain organic acids, which are sourced from natural products. They are stearic (octadecanoic) and 12-hydroxystearic (12-hydroxyoctadecanoic) acids [3]. Other industrial organic acids, such as those produced from oil refineries, tend to have shorter carbon chains and result in much lower viscosity. With an extra hydroxy group, 12-hydroxystearic acid produces better grease than stearic acid, which has markedly higher temperature resistance. For example, the 12-hydroxy stearic acid thickeners can increase the dropping point above 143 °C [3]. However, 12-hydroxy stearic acid is far more expensive than stearic acid. This is due to limited sources of 12-hydroxystearic acid. The only commercial source of 12-hydroxystearic acid is castor oil [4]. As a result, many grease producers have to rely on stearic acids, which can be produced from animal fats and vegetable oils. In addition to the lower thermo-resistance, using stearic acids might compete with food production and carry a significant cost. 

**Figure 1:**
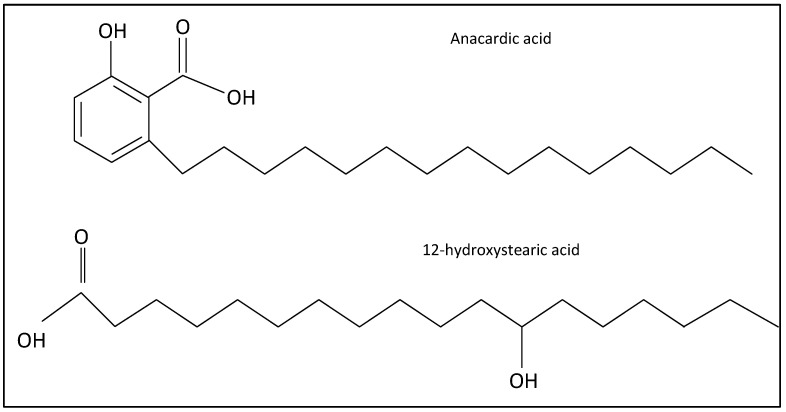
Structure of the natural-based fatty acids. Natural anacardic acids include four compounds with different saturation levels in the hydrocarbon chain.

Finding a new, economical, and environmentally friendly acid is important for the grease manufacturing industry. This study investigates alternative natural-based acids from the abundant agricultural waste, anacardic acids (Figure 1). In addition to long hydrocarbon chain, anacardic acid contains a bulky aromatic ring, which might increase viscosity. While anacardic acids are present in other plants [5], the compounds are abundant in cashew nut production [6]. The cashew tree (Anacardium Occidentale) is native to Brazil but is now cultivated in various tropical regions worldwide. The tree is an evergreen crop with a great tolerance against hot weather. Consequently, it is a valuable cash crop in many rural regions of Africa and Asia. It produces a fleshy fruit called the cashew apple, from which the cashew nut dangles. Cashew apples and nuts are naturally protected from insects and diseases by their allergen chemicals. The only edible part of the fruit is the kennel, with high nutrient and protein contents. The global cashew kernel production is around 700,000 metric tons annually [7]. The kennel (often referred to as “cashew nut”) is a highly valued dietary with many health benefits [8]. The kennel itself is surrounded by a double shell. The soft honeycomb matrix, in between the outer and inner shell, contains containing an allergenic phenolic liquid, which is known as the natural cashew nut shell liquid. 

Cashew shells, with a thickness between 3 and 4 mm, comprise a significant fraction (55–65% wt.) of cashew nuts. The most common technique of shelling is steam roasting [9], which is followed by the pressing of the steamed shells. While the solid part of the shells has a high thermal value, the liquor has very limited usage. It should be noted that resulted shells and pressed liquor have more water content than the natural shells. Cashew nut shell liquor on the whole has been investigated as the green reagent for coating resin [10] and base lubricants [11]. However, the specific components of liquor have different physical properties and can be used as specialized chemicals. The most well-known chemical that cashew nut shell liquor contains is cardanol, which can be converted into industrial chemicals such as extractants [12,13] and surfactants [14]. The molecular structure of natural cardanols offers comparable physio-chemical properties to petroleum-based organic reagents [15]. 

In addition to cardanol, natural cashew shell liquor contains four variations of anacardic acids, from saturated to tri-unsaturated [16]. The total anacardic acid fraction varies with growing regions and extraction methods. The reported value can be between wt. 50% [17] and wt. 90% [18]. While there are many proposed applications of anacardic acids [5], to the best of our knowledge, the application in lubricating grease has yet to be reported. As mentioned above, cashew fruits have high resistance to pest [5] and bacteria [19] due to a natural allergen. The allergenic properties are contributed to the biological properties of anacardic acids. On the other hand, such allergenic properties limit the application of CNSL in the food industry. Furthermore, this allergen poses problems during manual handling of CNSL as it tends to cause severe blisters in contact with human skin [20]. On contrast, castor oil-based compounds can be used for human ingestion and skin moisturizer. Consequently, utilizing CNSL in industrial applications, such as grease production, can save castor oil for more valuable applications. 

From the global cashew production, it can be estimated that the availability of anacardic acid is 500,000 metric tons per annum, which is approximately equal to castor oil production. However, castor oil has many other important food and industrial applications [4]. The current price of water-removed CNSL is around USD 300–400 per metric tonne, about 20% of the current castor oil price. Using this vast and affordable resource as a specialized chemical enhances socioeconomic benefits for developing countries in the tropical region. Due to its tolerance to hot and hash weather, it has been forecasted that cashew growth will increase in the following decades [21]. In addition, anacardic acid in grease production will save vegetable oils for other purposes, such as food production and direct human consumption. The use of natural-based compounds can reduce the carbon footprint of the industrial process from grease production and the end-of-life phase. 

Despite similar structure and physical properties (Figure 1), there is no study on the synthesis and stability of lithium anacardate. While anacardic acids can be extracted by chromatography [6] or supercritical CO_2_ [22,23], these processes are too costly for application, especially in rural areas. It is noteworthy that both anacardic and 12-hydroxystearic acids contain a hydroxyl group (Figure 1), which is not common amongst natural acids. From the molecular structure and reported physical properties, it is expected that anacardic acid can form stable lithium thickeners. This study aims to verify the capacity of cashew-based acid produced via a practical procedure as a precursor for grease thickeners. Instead of using solvent extraction, the study implements a common reaction followed by precipitation. Since the study focuses on grease thickener, the final product is lithium anacardate. Ultimately, the project aims to develop a sustainable and economical alternative to the current grease thickeners. A successful utilization of the CNSL in chemical products, instead of being used the fuel, can also reduce CO_2_ emission and improve the environmental and economic conditions of cashew-producing regions. 

## 2. Results and Discussion 

### 2.1. Separation of Anacardic Acid 

The first step in the process is the formation of calcium anacardate in acetone. Calcium is selected due to high molecular weight and ease of precipitation. Since CNSL is a mixture with high viscosity, dissolution in the solvent is necessary to facilitate the reaction. For this process, however, it is critical to have an aprotic solvent that lacks an acidic proton (does not have a hydrogen atom bonded to an atom of nitrogen or oxygen). The aprotic property prevents the solvent from reacting with a strong hydroxide as described below. While there are many solvents for dissolving CNSL, acetone was selected due to its availability, safety, and price. Due to its polar structure, acetone can dissolve a wide range of organic compounds in CNSL. The low viscosity and density also facilitate the precipitation process. The holding temperature and time, which significantly impact the industrial applications, were varied to identify the optimal ranges. 

**Figure 2:**
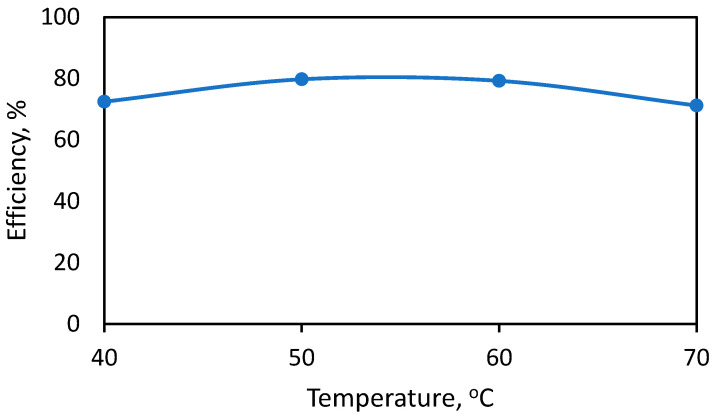
Effect of temperature on anacardic acid recovery efficiency.

The effect of temperature on the reaction of anacardic acid and calcium hydroxide is shown in Figure 2. In this figure, the reaction time was kept at 2 h. The results indicated a weak influence of temperature on efficiency. The optimal temperature is between 50 and 60 °C. At higher temperature, acetone can start evaporating (boiling point of acetone is 56 °C). 

Furthermore, the influence of reaction time was investigated. In this case, the separation of anacardic acid was obtained between 2 and 12 h at 50 °C. The results show that after 5 h, the extraction efficiency of anacardic acid was obtained the highest at 80.4% (based on the total mass of CNSL). 

**Figure 3:**
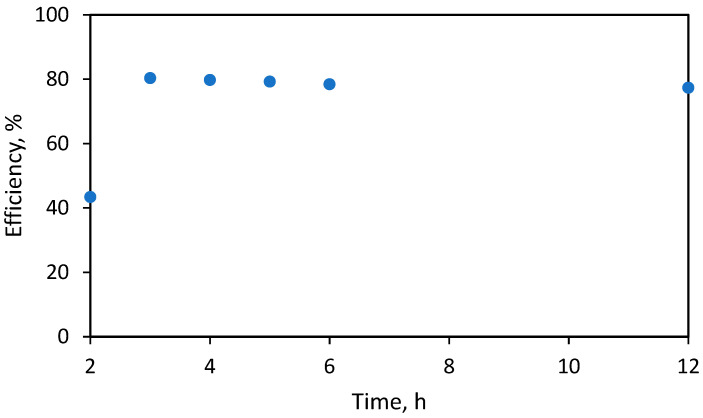
Effect of reaction time on anacardic acid recovery efficiency.

Increasing the reaction temperature or prolonging the reaction time can decrease the yield of anacardic acids (Figure 3). The underlying reason could be a side reaction, converting anacardic acid to cardol [24]. High temperatures might also decompose the acid molecules [25]. Anacardic acid tends to decarboxylate into cardanol at high temperature [20].

**Figure 4:**
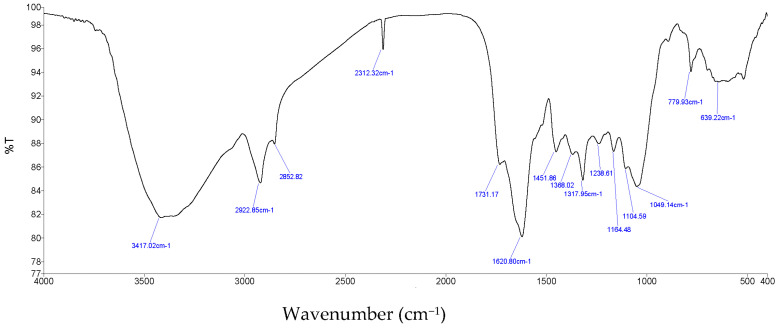
FT-IR spectrum of anacardic acid.

The obtained anacardic acids were analyzed by FTIR, ^13^C NMR and ^1^H NMR. In Figure 4, the peak at 1452 cm^−1^ is be shown as aromatic ring vibrations (including valence vibrations of C=C and strain vibrations of =C-H). Furthermore, peaks at 1621 cm^−1^ and 1731 cm^−1^ are typical for the C=O bond, whereas the peak at 2929 cm^−1^ is for a non-cyclic C-H bond. Finally, the 3417 and 2853 cm^−1^ peaks are the characteristics of the O-H bond.

**Figure 5:**
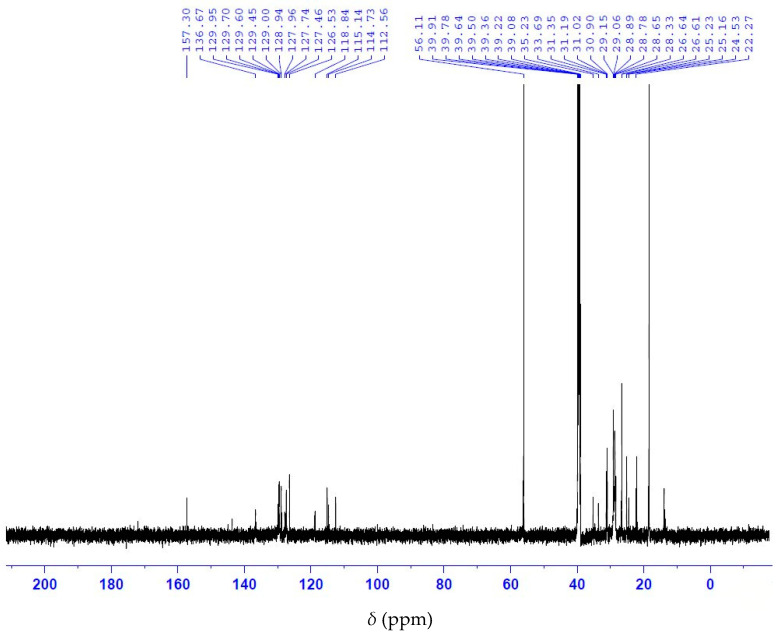
^13^C NMR spectrum of anacardic acid.

**Figure 6:**
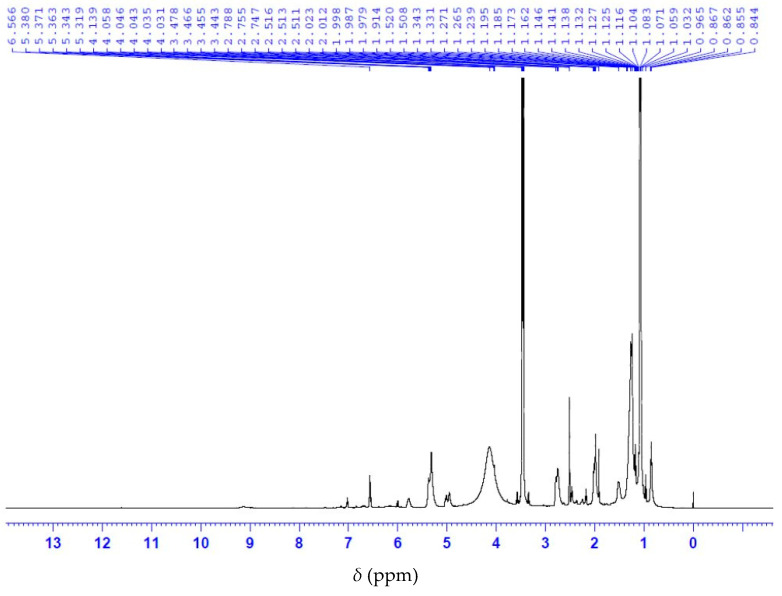
^1^H NMR spectrum of anacardic acid.

Analysis of the ^13^C NMR nuclear magnetic resonance spectrum of anacardic acid showed a signal display with a chemical shift at 175 ppm corresponding to the carbonyl group of the carboxylic acid (Figure 5). The ^1^H NMR spectrum shows the characteristic signals of the olefinic double bond at 4.8–6.0 ppm, as well as the H bonded to the aromatic ring at 6.5 to 7.5 ppm (Figure 6). Collectively, ^13^C and ^1^H NMR measurements indicate a signal of the anacardic acid moiety in the product [26]. It is important to note that the method does not require the full hydrogenation of anacardic acids. The process utilizes affordable reagents and simple heating equipment. Such process can be adapted to the production conditions in a rural area. In contrast, other acid separation processes [6,23] require high-pressure vessels and expensive reagents. 

### 2.2. Synthesis of Lithium Anacardate

**Figure 7:**
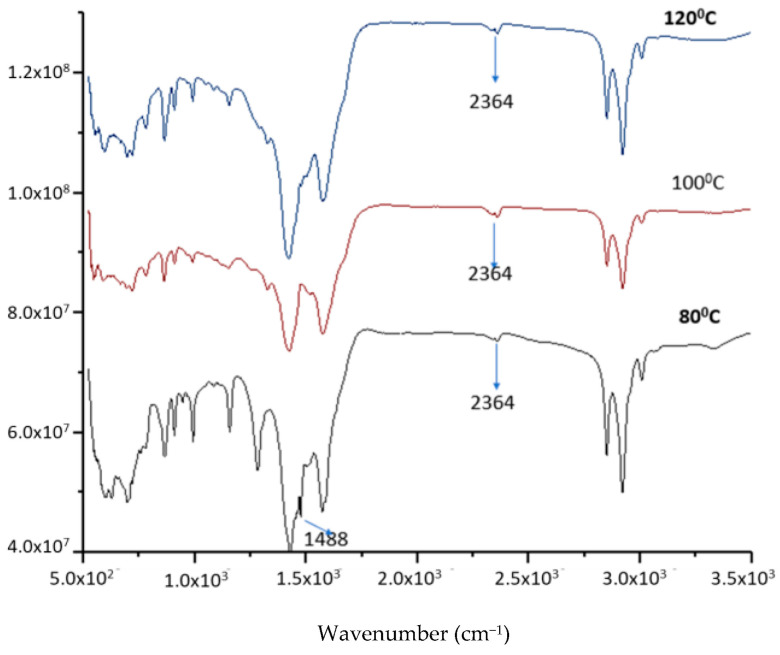
FTIR of the product from LiOH-anacardic acid reactions at different temperatures.

First, the effect of the reactant ratio and temperature on the product was investigated. Figure 7 shows the FTIR spectra of the lithium anacardate products at different temperatures. It can be seen that at 80 °C, the LiOH peak of 1488 cm^−1^ remains evident. This peak is completely removed at 100 and 120 °C. In addition, the lithium anacardate peak of 2364 cm^−1^ is more evident with increasing temperature. While higher temperatures can increase the reaction, they can also increase the decomposition of anacardic acids [24]. Consequently, 100 °C was selected as the optimal temperature for further experiments. 

**Figure 8:**
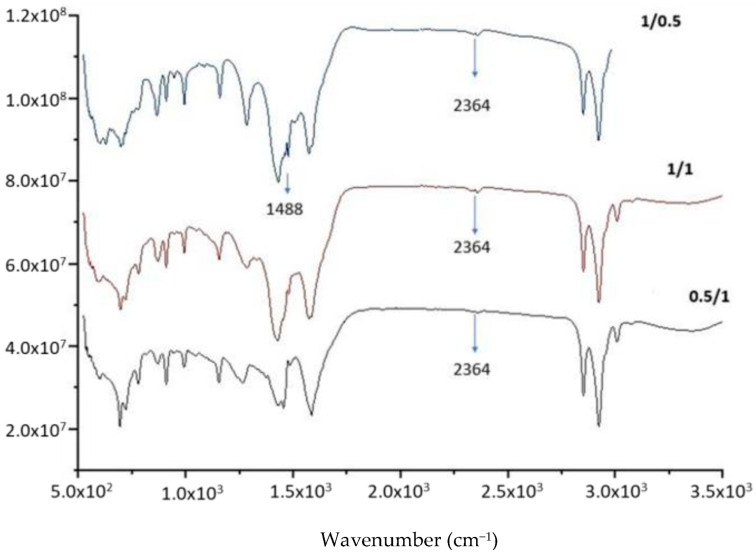
FTIR of the product from LiOH-anacardic acid reactions at different Li:anacardic molar ratios 0.5:1; 1:1; 1.5:1).

To confirm the reaction, the molar ratio of the two reactants was also varied from 1:2, 1:1 to 2:1. The peaks at 1488 and 2364 cm^−1^ (Figure 8) clearly confirm the conversion of LiOH to lithium anacardate. 

**Figure 9:**
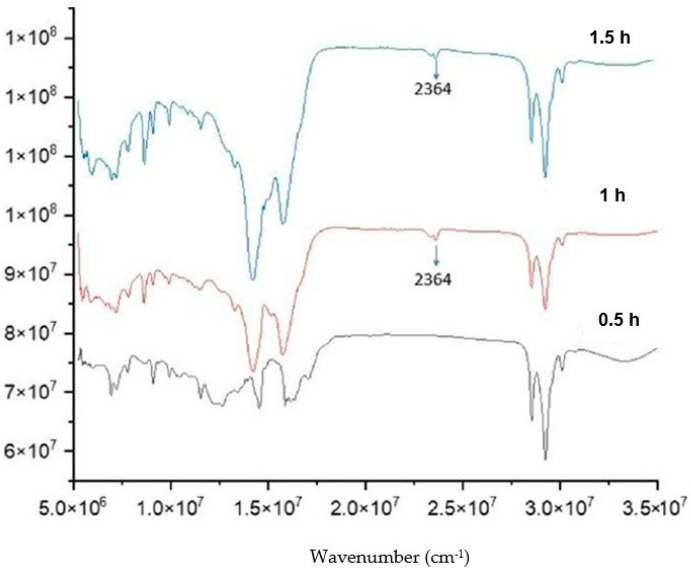
FTIR of the product from LiOH-anacardic acid reactions at 100 °C, lithium anacardate molar ratio 1:1 and different reaction times (0.5 h; 1 h; 1.5 h).

**Figure 10:**
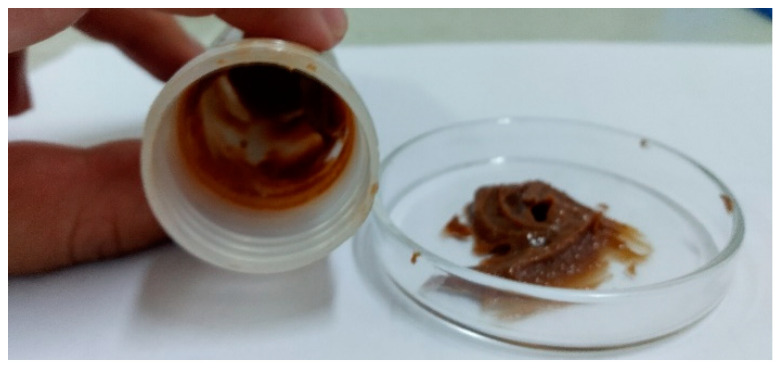
Grease obtained from lithium anacardate and paraffin oil.

Finally, the influence of reaction time was investigated (Figure 9). The spectra of the product after 0.5, 1 and 1.5 h are shown in Figure 10. It can be seen that the 1488 cm^−1^ disappeared after 1 h. Consequently, the required reaction for this process should be at least 1 h.

To validate the applicability of the lithium anacardate, the sample was mixed with different base oils. It was found that the production behaves as a gel (Figure 10). Further lubricating testing at high temperature (drop point, oxidation and penetration test [27]), is underway. 

In summary, lithium anacardate can be produced via an economical pathway. It should be noted that the procedure does not require the hydrogenation of anacardic acids [5]. The process also avoids the saponification step required for castor and vegetable oils [2]. The temperature requirements for the two reacting steps are relatively low, to prevent undesired chemical reactions. Consequently, the process is very competitive to other thickener productions. 

It also important to note the compatibility of the proposed process to current cashew processing. The most common cashew processing is slow steam roasting, up to a few hours, before cutting shell. The steam roasting softens the shell so that the processors can obtain the cashew kennels as whole, which is highly marketable. However, the slow steam also soaks the shell with water. In Vietnam, the steaming and shelling process are performed at the household levels. The shells are processed by small businesses in the local community. These businesses collect and cold press the shells in batch processes. Upon cold-pressing, the water content of CNSL can be up to 50% by weight. This CNSL is often referred to as a “raw” CNSL. Subsequently, the processing facilities have to remove water from the raw CNSL by distilling at 110–120 °C. In rural areas, distillation is performed in batch boilers, which is fuelled by local biomass (in most cases, the compressed cashew shells). The obtained liquor is then marketed as “processed” or “water-free” CNSL. The processed CNSL is the raw feedstock for our proposed synthesis of lithium anacardate, as described in Section 3. Since the distilled CNSL is heated, the current distillation can be directly combined with our first step, that is, producing calcium anacardate at an elevated temperature, 50 to 60 °C. In this step, the only required reagent is acetone. The step can be performed in existing facilities with a minimal modification. The solid precipitate, calcium anacardate, can be easily transported to the centralized plant to process to lithium anacardate (Steps 2 and 3). The remaining organics from CNSL can be disposed of by the current practice (mixed with diesel for combustion-based power plants).

In our process, calcium hydroxide can be regenerated from the second step. As a result, lithium hydroxide is a required consumable. Lithium demands and prize have skyrocketed recently due to high demand of lithium-ion batteries. Due to the short life cycle of battery (between 7 and 10 years), there will be a huge quantity of the spent batteries. While there are a lot of efforts to recycle the spent batteries, only the most expensive metals, such as cobalt and nickel, are economically recyclable. Lithium, due its reactivity, is the last metal to be recovered in the hydrometallurgical process [28]. As a result, lithium hydroxide from the recycling has a high level of impurities and is not suitable for battery feedstock [29]. Currently, the source for battery-grade lithium feedstock is the virgin lithium source, such as brines and spodumene [30]. Hence, the recycled lithium hydroxide from the spent batteries can be used in the grease production. Such combination will significantly improve the sustainability of energy transformation. 

In addition to economic and technical advantages, the process also reduces the environmental impact of cashew nut production. While CNSL has other potential applications, such as antioxidants [31], surfactants [12] or pharmaceutical usage [32], those processes are too complicated for developing regions. The proposed process in this study can be applied with simple equipment and is thus fully utilized in the sustainable development in the cropping regions [13]. It should be noted that cashew nut shell liquor can be used as a base oil for lubricant as well [33]. It is expected that the lithium anacardate is highly miscible with CNSL due to similarity in hydrocarbon structure. Consequently, CNSL can be used to provide both components of grease. Further investigation with the anacardate-based grease is conducted. 

## 3. Experiment

### 3.1. Materials

Cashew nut shell liquor was obtained from a producer in Binh Phuoc Province (Vietnam). Reagents calcium hydroxide, lithium hydroxide and chloric acid were obtained from Merck (Hochiminh City, Vietnam). Organic solvents, acetone (99%), alcohol (ethanol 99%) and cyclohexane, (99.5%) were obtained from Merck.

The synthesis and separation procedure contained three steps: (i) forming and separating calcium anacardate, (ii) forming and separating anacardic acid, (iii) forming lithium anacardate.

### 3.2. Forming and Extracting Calcium Anacardate from Cashew Nut Shell Liquor

Cashew nut shell liquor (50 g) was dissolved in 300 mL acetone. Subsequently, 60 g of calcium hydroxide was added to the mixture with continuous stirring. The solution was heated and maintained at a designated temperature and period. In this study, the temperate was selected at either 40, 50, 60 or 70 °C. These temperatures were selected based on the boiling point of acetone (56 °C). The reaction time was up to 12 h. After the reaction, a precipitate appeared. The solid precipitate should mostly contain calcium anacardate. The precipitates were recovered, filtered and then washed with 160 mL of acetone. The solids were left to dry naturally for 2 h to obtain calcium anacardate [34].

### 3.3. Forming and Separating Anacardic Acids

While calcium helps to precipitate acidic content, it is necessary to protonate the acid before forming lithium anacardate. Direct replacement of calcium with lithium would complicate the separation process. Hence, neutralization with a strong acid was applied. 

The obtained calcium anacardate from the previous step was dissolved in a strong acid solution (250 mL of aqueous solution of HCl at a concentration of 81 g/L). The mixture was stirred continuously for 30 min at room temperature (25 °C). The aqueous solution was transferred to an extraction column and extracted with 300 mL of cyclohexane. The organic layer was separated and washed with 200 mL of distilled water in the extraction column. The obtained solid was dried with anhydrous sodium sulphate to obtain the final product, anacardic acid.

### 3.4. Synthesis of Lithium Anacardate 

The obtained anacardic acid was dissolved in pure ethanol (20 g of anacardic acid in 50 mL of ethanol). The alcohol solution was gently heated to 80 °C. A solution of lithium hydroxide was prepared by mixing LiOH·H_2_O with water. The quantity of LiOH·H_2_O was selected to obtain the molar ratios of 1:2, 1:1 and 2:1 to anacardic acid. The aqueous solution was added slowly to the ethanol solution at 80 °C. The mixture was heated and maintained at 100 °C for 1 h to ensure a complete reaction. The heating was maintained for another hour to remove the water until the solution turned brown. The remaining solution was pulverized and dried at 65 °C for 30 h. The final product recovered was lithium anacardate.

### 3.5. Characterization

The immediate and final products were characterised by FT-IR (Fourier-transform infrared spectroscopy), proton and carbon NMR (nuclear magnetic resonance). IR spectra were collected by a Nicolet Impact iS10 FTIR Spectrometer (Thermo Fisher Scientific, Waltham, MA, USA). Measuring conditions: resolution 0.4 cm^−1^ (varies from 64 to 0.4 cm^−1^); the 1 min signal-to-noise ratio: 35,000:1; Wavelength Accuracy: 0.01 cm^−1^ at 2000 cm^−1^; Scan rate: 40 times/s. 

^1^H NMR spectra were recorded at 298 K using a Bruker Avance III 500 MHz spectrometer. Data were expressed in parts per million (ppm) downfield shift from tetramethylsilane with residual solvent as an internal reference (δ 7.26 ppm for chloroform) and was reported as position (δ in ppm), multiplicity (s = singlet, d = doublet, t = triplet, q = quartet, m = multiplet), coupling constant (J in Hz) and integration (number of protons). ^13^C NMR spectra were recorded at 298 K using a Bruker Avance III 125 MHz spectrometer (Bruker, MA, USA) with complete proton decoupling. Data were expressed in parts per million (ppm) downfield shift relative to the internal reference (77.2 ppm for the central peak of deuterated chloroform) and is reported as the shift position (δ in ppm).

### 3.6. Grease Production

The grease was prepared following the industrial guidelines, with paraffin as the base oil [2]. The weight ratio between lithium anacardate and base oil was selected at 16–84%. First, 8 g of lithium anacardate was mixed in 20 g of ethanol. Second, 42 g of paraffin oil was deposited in a glass container. The oil was heated to 80 °C and stirred via magnetic stirred at 500 rpm. The lithium anacardate/ethanol was added gradually to the heated oil. The mixture was heated to and maintained at 150 °C for 1 h. The mixture was allowed to cool to the room temperature to obtain a lubricant grease.

## 4. Conclusions

Anacardic acid was separated from the cashew nut shell liquor by the precipitation method. The obtained anacardic acid was reacted with LiOH to form lithium anacardate, which is a thickener for lubricating grease. It was found that the recovery efficiency was around 80%. The optimal reaction temperature and time conditions were 100 °C and 1 h, respectively. The method provided an economical alternative to castor and other vegetable oils. The process did not require a hydrogenation step. Consequently, the obtained lithium anacardate contained all four different carbon chains of anacardic structure. Having various numbers of double carbon bonds might improve the functionality of the resulting grease. The procedure presented a simple pathway to produce the precursor for the lubricating grease from agriculture waste. The alternative might help to reduce the industry’s reliance on castor and vegetable oil. 

From the molecular structure, the CNSL-based thickener is expected to have much higher temperature resistance than stearic-based thickener. It may have a comparable performance to 12-hydroxystearic-based thickener. In addition to cleaning surfactants [14] and metallurgically extractants [12], making lubricating thickeners will enhance the CNSL as a sustainable feedstock for green chemistry. The proposed grease production can be also combined with recycled lithium from the spent batteries to improve the sustainability of the battery industry. 

## Data Availability

All data available in the manuscript.
